# Evaluation of Humoral and Cellular Responses in SARS-CoV-2 mRNA Vaccinated Immunocompromised Patients

**DOI:** 10.3389/fimmu.2022.858399

**Published:** 2022-03-22

**Authors:** Matthijs Oyaert, Marie-Angélique De Scheerder, Sophie Van Herrewege, Guy Laureys, Sofie Van Assche, Melissa Cambron, Leslie Naesens, Levi Hoste, Karlien Claes, Filomeen Haerynck, Tessa Kerre, Steven Van Laecke, Wim Van Biesen, Peggy Jacques, Bruno Verhasselt, Elizaveta Padalko

**Affiliations:** ^1^ Department of Laboratory Medicine, Ghent University Hospital, Ghent, Belgium; ^2^ Department of General Internal Medicine, Ghent University Hospital, Ghent, Belgium; ^3^ Department of Neurology, Ghent University Hospital, Ghent, Belgium; ^4^ Department of Neurology, Algemeen Ziekenhuis (AZ) Sint-Jan Brugge Oostende, Bruges, Belgium; ^5^ Department of Internal Medicine and Pediatrics, Ghent University Hospital, Ghent, Belgium; ^6^ Primary Immunodeficiency Research Lab, Ghent University, Ghent, Belgium; ^7^ Department of Haematology, Ghent University Hospital, Ghent, Belgium; ^8^ Department of Nephrology, Ghent University Hospital, Ghent, Belgium; ^9^ Department of Rheumatology, Ghent University Hospital, Ghent, Belgium

**Keywords:** SARS-CoV-2, immunocompromised, antibodies, vaccination, humoral response

## Abstract

**Background:**

Immunocompromised patients are at increased risk of severe COVID-19 and impaired vaccine response. In this observational prospective study, we evaluated immunogenicity of the BNT162b2 mRNA vaccine in cohorts of primary or secondary immunocompromised patients.

**Methods:**

Five clinical groups of immunocompromised patients [primary immunodeficiency (PID) (n=57), people living with HIV (PLWH) (n=27), secondary immunocompromised patients with a broad variety of underlying rheumatologic (n=23) and homogeneous (multiple sclerosis) neurologic (n=53) conditions and chronic kidney disease (CKD) (n=39)] as well as a healthy control group (n=54) were included. Systemic humoral and cellular immune responses were evaluated by determination of anti-SARS-CoV-2 Spike antibodies using a TrimericS IgG assay (Diasorin) and through quantification of interferon gamma release in response to SARS-CoV-2 antigen with QuantiFERON SARS-CoV-2 assay (Qiagen), respectively. Responses were measured at pre-defined time-points after complete vaccination.

**Results:**

All healthy controls, PLWH and CKD-patients had detectable antibodies 10 to 14 days (T2) and 3 months (T3) after administration of the second vaccination. In contrast, only 94.5% of the PID, 50.0% of the rheumatologic and 48.0% of neurologic patients developed antibodies at T2 and only 89.1% of the PID, 52.4% of the rheumatologic and 50.0% of neurologic patients developed antibodies at T3. At T3 no significant differences in cellular response between the healthy control group and the PLWH and CKD groups were found, while proportions of reactive subjects were lower in PID and rheumatologic patients and higher in neurologic patients. Humoral and cellular immune responses significantly correlated in the healthy control, PID, PLWH groups for all 3 antigens.

**Conclusion:**

Patients with acquired or inherited immune disorders may show variable immune responses to vaccination with the BNT162b2 mRNA vaccine against SARS-CoV-2. Whether humoral, cellular or both immune responses are delayed depends on the patient group, therapy and individual risk factors. These data may guide the counselling of patients with immune disorders regarding vaccination of SARS-CoV-2.

## Introduction

After vaccination, most individuals mount a robust humoral immune response against severe acute respiratory syndrome coronavirus 2 (SARS-CoV-2), with antibody levels peaking approximately 4-8 weeks after full vaccination (1, 2). Vulnerable populations such as the elderly, immunocompromised or those suffering from chronic underlying diseases requiring continuous medical interventions are at risk of severe COVID-19 disease and associated mortality (3, 4). Immunocompromised patients are a particular high-risk group as immunosuppression has been identified as a key risk factor for clinically severe COVID-19 (5). A hospital environment, regularly visited for therapeutic interventions, is dangerous for these patients.

Serological assays for virus-specific antibody response are commonly employed to establish the immunological response to vaccines and as a biomarker for protection against infection in general (6). While official policy recommendations do not include antibody testing as part of the vaccination programs, serological surveillance has been included in several pilot settings, and many studies have already established the extent and magnitude of antibody response following administration of COVID mRNA vaccines (7–9). These studies, mostly performed in selected patient groups, have demonstrated an impaired humoral immune response in kidney transplant recipients (10), patients on renal replacement therapy (11) and after solid organ and lung transplantation (12, 13), patients living with human immunodeficiency virus (HIV)-1 infection (HIV, PLWH) (14–16), multiple sclerosis (MS) (17, 18) and diagnosed with rheumatoid arthritis (19, 20), spondyloarthritis (21), or inborn errors of immunity (22, 23). While antibody responses have already been characterized within vaccinated immunocompromised patients, little is known on the ensuing cellular immune response of vaccine induced antibodies in patients diagnosed with rheumatologic disorders (e.g. rheumatoid arthritis, systemic lupus erythematosus), multiple sclerosis (MS), primary immune deficiency (PID) or chronic kidney disease (CKD).

To assess immunogenicity of SARS-CoV-2 vaccination in different clinical groups of immunocompromised patients, we characterised humoral immune responses in individuals after 2 administrations of the BNT162b2 vaccine; with a median 4 week interval, using a recently developed commercial immunoassay for the quantification of antibodies against the Trimeric complex. Considering cellular immunodeficiency in our patient population, we also evaluated cellular response by means of a QuantiFERON assay.

## Materials and Methods

### Study Population

This observational prospective study included 5 clinical groups of immunocompromised patients as well as a healthy control group. The patients were categorized into groups of which 57 patients were diagnosed with primary immunodeficiency (PID), 27 PLWH with CD4 count at the moment of inclusion below 350 per µL, 53 patients with homogeneous [MS receiving mono B-cell depletion therapy (BCDT)] neurologic and 23 patients with diverse rheumatic disorders requiring immunosuppressive treatment and 39 patients with CKD [KDIGO G3b: n=9 (23.0%); G4: n=26 (66.7%); G5: n=4 (10.3%)]. A summary of the patient characteristics, including their initial diagnosis and therapy is presented in **Table 1**. Adults aged 17 to 63 years (median: 37 years; female: n = 23; male: n=31), without known medical conditions, were recruited as healthy controls. All subjects received two doses of the BNT162b2 mRNA COVID-19 vaccine (Pfizer-BioNTech, Mainz, Germany), with a median 28 days interval (range: 20 to 42 days). Humoral immune response was evaluated prior to vaccine administration (T0), 21 to 28 days after administration of the first vaccine dose (T1), 10 to 14 days (T2) and 3 months (T3) after administration of the second vaccine dose. The cellular response was evaluated at T0 and T3.

**Table 1:** Characteristics of the different patient groups included in the study.

	Primary immunodeficiency		Patients living with HIV		Neurology		Rheumatology		Chronic kidney disease	
***Number***	*57*		*27*		*53*		*23*		*39*	
***Age (y)***	*21 (15-60), p-value<0.001*		*47 (30-66), p-value<0.001*		*40 (21-74), p-value=0.447*		*53 (20-83), p-value<0.001*		*43 (19 - 65), p-value=0.177*	
***Gender (F/M)***	*22/35*		*8/19*		*36/17*		*15/8^d^*		*20/19*	
***Number of patients included at:***										
*** T0***	*57*		*27*		*53*		*23*		*39*	
*** T1***	*57*		*27*		*53*		*22*		*39*	
*** T2***	*55*		*23*		*50*		*20*		*39*	
*** T3***	*55*		*25*		*50*		*21*		*36*	
***Lymphocyte count^a^***										
*** Total count***	*1755 (370 – 840)*		*NA*		*NA*		*NA *		*1.7 (0.8 – 5.8)*	
*** CD19+B-cell count***	*755 (198 – 390)*		*NA*		*NA*		*NA*		*NA*	
*** CD4+T-cell count***	*241 (0 – 698)*		*254 (128 - 346)*		*NA*		*NA*		*NA*	
***Months between BCDT and T0^b^***	*NA*		*NA*		*2.5 (1.6)*		*4.6 (1.9)*		*NA*	
***Disease (characteristics)***	***AB deficiency***	*32 (56.1%)*	***Y living with HIV***	*8.1 (0.5 – 28.7)*	***Multiple sclerosis***	*51 (96.2%)*	***Rheumatoid arthritis***	*11 (47.8%)*	Inherited kidney disease***	*11 (28.2%)*
	Combined IDs*	*12 (21.1%)*	***Y receiving therapy***	*6 (0-19)*	***Neuromyelitis optica***	*2 (3.8%)*	***Systemic sclerosis***	*3 (13.0%)*	***Glomerulonephritis***	*9 (23.1%)*
	Others**	*13 (22.8%)*					***Systemic lupus***	*3 (13.0%)*	***CAKUT***	*8 (20.5%)*
							***Psoriatic arthritis***	*2 (8.7%)*	***Interstitial nephritis***	*4 (10.3%)*
							***ANCA vasculitis***	*1 (4.3%)*	***Diabetic nephropathy***	*3 (7.7%)*
							***GPA***	*1 (4.3%)*	***Nephrectomy***	*2 (5.1%)*
							***IgA dermatosis***	*1 (4.3%)*	***CKD after AKI***	*1 (2.6%)*
							***Polymyositis***	*1 (4.3%)*	***Multiple myeloma***	*1 (2.6%)*
***Medication***	***IRT***	*30 (52.6%)*			***OCRE***	*50 (94.3%)*	***RITUX***	*5 (21.7%)*	***No IS medication***	*31 (79.5%)*
	***IRT + RITUX***	*3 (5.3%))*			***RITUX***	*3 (5.7%)*	***RITUX + DMARD***	*6 (26.1%)*	***CORTIC***	*4 (10.3%)*
	***IRT + CORTIC***	*3 (5.3%)*					***RITUX + DMARD + CORTIC***	*4 (17.4%)*	***CORTIC + DMARD + belimumab***	*1 (2.6%)*
	***IRT + IS***	*2 (3.5%)*					***RITUX + CORTIC***	*1 (4.3%)*	***CORTIC + bortezomib***	*1 (2.6%)*
	***IRT + CORTIC + IS***	*1 (1.8%)*					***Methotrexate***	*4 (17.4%)*	***CORTIC + DMARD***	*1 (2.6%)*
	***IRT + Tocilizumab***	*1 (1.8%)*					***DMARD + belimumab***	*2 (8.6%)*	***CORTIC + DMARD + IS***	*1 (2.6%)*
	***No IRT***	*16 (28.1%)*					***Adalimumab***	*1 (4.3%)*		
	***CORTIC + DMARD***	*1 (1.8%)*								

Results are presented as median (range), unless otherwise specified. Significant difference in ages between the patient group and the healthy control group are indicated. ^a^at inclusion; ^b^mean (standard deviation),^c^According to the IUIS classification, ^d^Disease activity score (DAS28): moderate disease (n=1), low disease activity (n=13), remission (n=9) *of which 8 combined immunodeficiency with associated or syndromal features and 4 immunodeficiency affecting cellular and humoral immunity; **of which 2 defect in intrinsic and innate immunity, 4 immunodeficiency affecting cellular and humoral immunity, 3 phenocopy of inborn errors of immunity, 3 complement deficiency, 2 defects in intrinsic and innate immunity and 1 congenital defect of phagocyte number or function; *** of which 8 patients had polycystic kidney disease, 1 patient with autosomal dominant tubule-interstitial kidney disease and 1 patient with Alport disease. Any of the CKD patients received renal replacement therapy.

NA, not applicable; GPA, granulomatosis with polyangiitis; RITUX, Rituximab; DMARD, Disease modifying anti-rheumatic Drug; CORTIC, Corticosteroid; METHO, Methotrexate; OCRE, ocrelizumab; IRT, Immunoglobulin replacement therapy; Y, Years; ID, immune deficiency, AB, antibody; GFR, Glomerular Filtration Rate; CKD, chronic kidney disease; AKI, acute kidney disease; CAKUT, Congenital Abnormalities of the kidney and urinary tract; IUIS, International Union of Immunological Societies; IS, immune suppression.

Previous infection was defined as IgG anti-nucleocapsid (N) positivity at T0, IgG anti-spike (S) positivity before vaccination, and/or a history of positive polymerase chain reaction (PCR) result on nasopharyngeal swab. At each visit, the subject was questioned whether COVID-19 compatible symptoms were present between two study visits.

### SARS-CoV-2 Humoral Immune Response

Humoral immune response was evaluated at each time point using the Liaison^®^ SARS-CoV-2 TrimericS IgG chemiluminescent immunoassay (CLIA) on the Liaison XL (Diasorin S.P.A., Saluggia, Italy) according to the manufacturer’s instructions and using the manufacturer’s cut-off for positivity of 33.8 binding activity units (BAU)/mL. This assay quantitatively determines antibodies against the TrimericS complex, which includes the Receptor Binding Domain (RBD) and N-terminal domain (NTD) sites including S1 and S2. Samples above the measuring range of 2000 BAU/mL for anti-S were further diluted 1:10 using the Diasorin assay manual diluent to achieve exact analytical measurement. The assay is correlated with the micro-neutralization and standardized against the WHO internal standard (NIBSC 20-136) (24).

The quantitative detection of serum IgG antibodies to the nucleocapsid (N) protein of SARS-CoV-2 was performed on all subjects positive for IgG anti-S1 at T0, using a chemiluminescent micro-particle immunoassay (CMIA) on the Abbott Architect i2000SR analyser using the manufacturer’s cut-off for positivity of 1.4 S/CO (SARS-CoV-2 IgG Quant assay, Abbott, Lake Forest Illinois, USA).

### SARS-CoV-2 Cellular Immune Response

Cellular immune response was assessed by measuring the secretion of interferon(IFN)-gamma by peripheral blood lymphocytes upon SARS-CoV-2 glycoprotein stimulation using the QuantiFERON SARS-CoV-2 research only assay (Qiagen). The QuantiFERON SARS-CoV-2 assay evaluates three antigen (Ag) tubes, i.e. Starter (Antigen 1 and 2) and Extended (Antigen 3) blood collection tubes, that use a combination of proprietary antigen peptides specific to SARS-CoV-2 to stimulate lymphocytes involved in cell-mediated immunity in heparinized whole blood samples. The QuantiFERON SARS-CoV-2 Ag1 tube contains CD4+ epitopes derived from the S1 subunit (RBD) of the spike protein, the Ag2 tube contains CD4+ and CD8+ epitopes from the S1 and S2 subunits of the Spike protein, and the Ag3 tube consists of CD4+ and CD8+ epitopes from S1 and S2, plus immunodominant CD8+ epitopes from whole genome. The tubes were gently mixed with the whole blood to re-solubilize the content coated onto the inner walls. QuantiFERON Nil and Mitogen blood collection tubes were used as negative and positive controls, respectively. IFN gamma was measured by CLIA on the Liaison XL (Diasorin). Elevated response was defined as a value at least 0.15 IU/mL greater than the background IU/mL value from the QuantiFERON SARS-CoV-2 Nil tube (25). The Nil tube value was subtracted to mitigate against background IFN gamma in the sample that was not a result of SARS-CoV-2 specific T-cell stimulation. The cellular response was evaluated at T0 (Ag1 and Ag2) and T3 (all antigens).

### Statistical Analysis

Humoral and cellular immune responses were compared at each time point between the healthy control and immunocompromised patient groups and between the different time points within one patient group. Statistical differences were assessed using non-parametric Mann-Whitney U test. All tests were two-sided and statistical significance was defined as a p-value < 0.05. Correlations between humoral and cellular immune responses and other available variables were assessed by means of Spearman rank correlation coefficient (*r)*. Results are presented as median and range, unless the results in the dataset are normally distributed. In that case, results are presented as mean and standard deviation (SD). Normality of the dataset was tested by means of the D’Agustino-Pearson test. Analyses were performed using Microsoft Excel software, Medcalc statistical software (version 15.6.1.) and Prism (version 9.0, Graphpad software). This prospective study was performed at the Ghent University Hospital after approval by the local ethics committee (B6702021000426). All participants signed informed consent prior to inclusion in the study.

## Results

### Subjects

**Figure 1:**
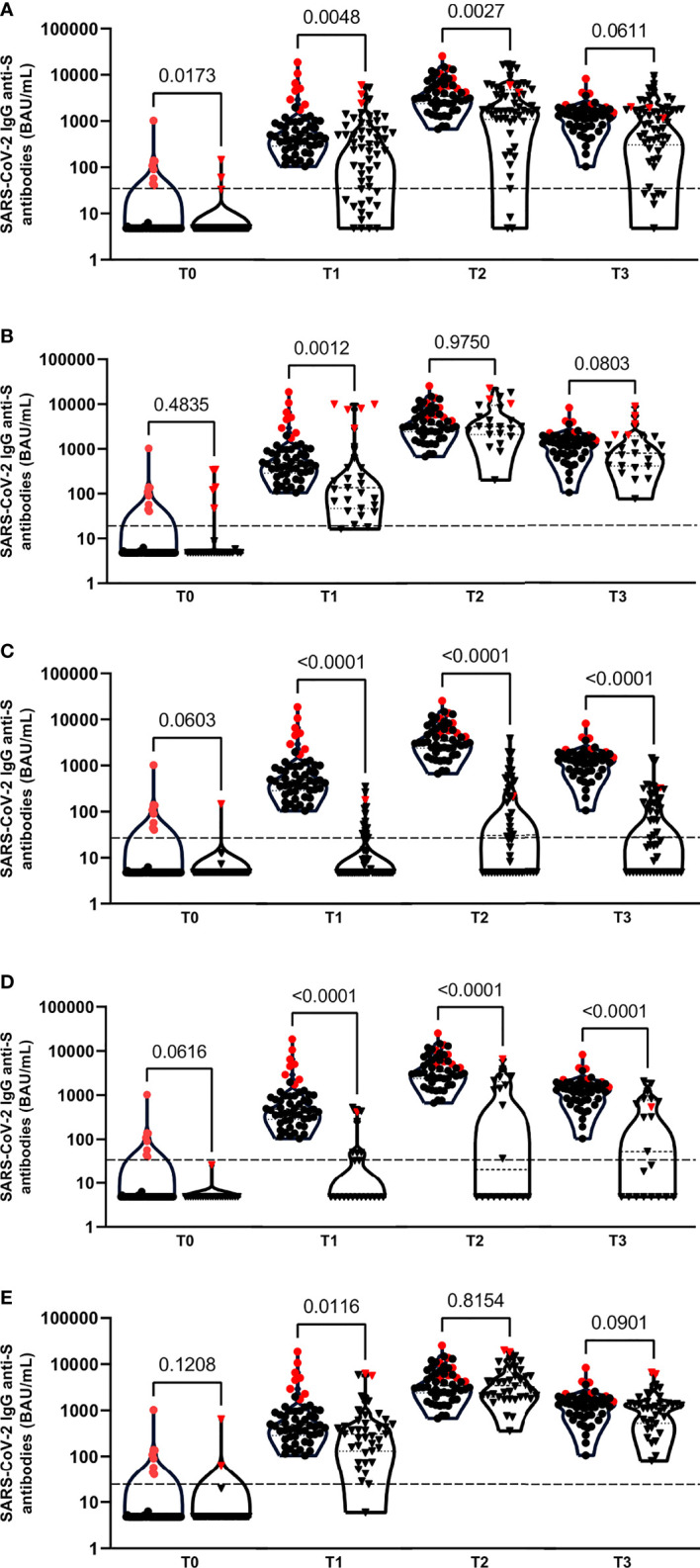
Violin plots of anti-SARS-CoV-2 IgG antibodies at T0, T1, T2 and T3 time points between the healthy control group (●) and patient groups (▼): primary immunodeficiency patient group **(A)**, PLWH **(B)**, neurologic **(C)**, rheumatologic **(D)** and CKD **(E)** patient groups. Patients with a documented Sars-CoV-2 infection are indicated with red symbols. Each plot represent the median, 25th and 75th percentiles. Outliers were determined by 1.5 time IQR. Statistical significance was calculated by Mann-Whitney U test. Significance was defined as a p-value < 0.05. Each plot represent the median, 25th and 75th percentiles. Outliers were determined by 1.5 time IQR. Statistical significance was calculated by Mann-Whitney U test. Significance was defined as a p-value < 0.05.

In total, 253 subjects who received two doses of the BNT162b2 mRNA COVID-19 vaccine were enrolled in this study. Characteristics of the different patient groups are summarized in **Table 1**. Twenty out of these 253 subjects (7.9%) had a positive IgG anti-S1 positive reaction at T0, of which nine patients (3.6%) were SARS-CoV-2 IgG anti-N positive. These previously infected patients had higher antibody concentrations in the upper quartile of the plots at each study visit compared to the previously non-infected patients (**Figure 1**). None of the patients declared having complaints compatible to COVID-19 between the different study visits.

### Humoral Immune Response

All healthy controls, PLWH and CKD patient groups had detectable antibodies against the SARS-CoV-2 RBD of the spike protein 10 to 14 days (T2) and 3 months after administration of the second vaccine dose (T3) (**Table 2**). In contrast, only 94.5% of the PID (range antibody concentration: <4.81 – 17.000 BAU/mL), 50.0% of the rheumatologic (range: <4.81 – 6610 BAU/mL) and 48.0% of neurologic patients (range: <4.81 – 3890 BAU/mL) had positive antibodies at T2; and only 89.1% of the PID (range: <4.81 – 9550 BAU/mL), 52.4% of the rheumatologic (range: <4.81 – 2080 BAU/mL) and 50.0% of neurologic patients (range: <4.81 – 1480 BAU/mL) developed antibodies at T3 (**Table 2**). The average time to positive result was slower in earlier samples post-first dose (T1) in all the patient groups compared to the healthy control group (**Figure 1**) (*p-value* T1<0.05). Significantly lower median antibody concentrations were found in the PID (T2), neurologic (T2 and T3) and the rheumatologic (T2 and T3) patients groups as compared to the healthy control group. For the PLWH and CKD patient groups, no statistical differences compared to the healthy control group were observed at T2 and T3 in humoral cell response (**Figure 1**). The distribution of the humoral immune response within each patient group at each time point is presented in **Table 2**.

**Table 2:** IgG anti-S1 antibody positivity (cut-off: 33.8 BAU/mL) at baseline (T0), at the time of second vaccine dose administration (T1), 10-14 days after administration of the second dose (T2) and 3 months after administration of the second vaccine dose (T3) for each patient group. Results are presented as number of positive patients per total number of patients. At each time point, also the median (min-max) IgG anti-S1 antibody concentration (BAU/mL) in the different patients groups is described.

Patient group	T0		T1		T2		T3	
	Positivity	Median (BAU/mL, min-max)	Positivity	Median (BAU/mL, min-max)	Positivity	Median (BAU/mL, min-max)	Positivity	Median (BAU/mL, min-max)
**Healthy controls**	8/54	<4.81	54/54	495	52/52	3455	51/51	1320
	(14.8%)	(<4.81 – 1020)	(100%)	(105 – 18700)	(100%)	(674 – 25400)	(100%)	(104 – 8330)
**PID**	2/57	<4.81	43/57	307	52/55	1690	49/55	941
	(3.5%)	(<4.81 – 144)	(75.4%)	(<4.81 – 5960)	(94.5%)	(<4.81 – 17100)	(89.1%)	(<4.81 – 9550)
**PLWH**	5/27	<4.81	23/27	136	23/23	3140	25/25	788
	(18.5%)	(<4.81 – 348)	(85.2%)	(16.4 – 9930)	(100%)	(200 – 22400)	(100%)	(75.4 – 8860)
**Neurology **	1/53	<4.81	10/53	<4.81	24/50	30.7	24/50	28.1
	(1.9%)	(<4.81 – 146)	(18.9%)	(<4.81 – 353)	(48.0%)	(<4.81 – 3890)	(48.0%)	(<4.81 – 1480)
**Rheumatology **	0/23	<4.81	7/22	<4.81	10/20	20.9	11/21	<4.81
	(0%)	(<4.81 – 26.2)	(31.8%)	(<4.81 – 536)	(50.0%)	(<4.81 – 6610)	(52.4%)	(<4.81 – 2080)
**CKD**	2/39	<4.81	37/39	313	39/39	3450	36/36	1115
	(5.1%)	(<4.81 – 647)	(94.9%)	(6 – 6300)	(100%)	(361 – 19800)	(100%)	(78.8 – 6550)

PID, primary immunodeficiency; PLWH, patients living with HIV; CKD, chronic kidney disease.

Separate analysis of the humoral immune response within the PID patient group revealed that humoral responses are delayed within each PID subgroup (primary antibody deficiency, combined immunodeficiency and others) to the same extent. A reduced seroconversion rate was observed in patients receiving immunoglobulin replacement therapy (IRT) (n=40) as compared to patients not receiving this therapy (*p-value <0.05* at T1 and T2; **Supplementary Figure 1A**). A significant correlation [*r* = 0.438 (*p-value* < 0.001)] was observed between the total B-cell count at time of inclusion (T0) and SARS-CoV-2 IgG anti-S1 antibodies at T3.

Within the rheumatologic patient group, statistical differences in humoral immune responses were observed at T2 and T3 between the group of patients receiving BCDT (n=16) compared to the group that did not (*p-value <0.05* at T1, T2 and T3; **Supplementary Figure 1B**). All patients in the neurologic patient group received BCDT (**Table 1**). As only 5 patients in the other patient groups received BCDT without other concomitant therapies, humoral response across the different patient groups was not assessed.

### Cellular Immune Response

**Figure 2:**
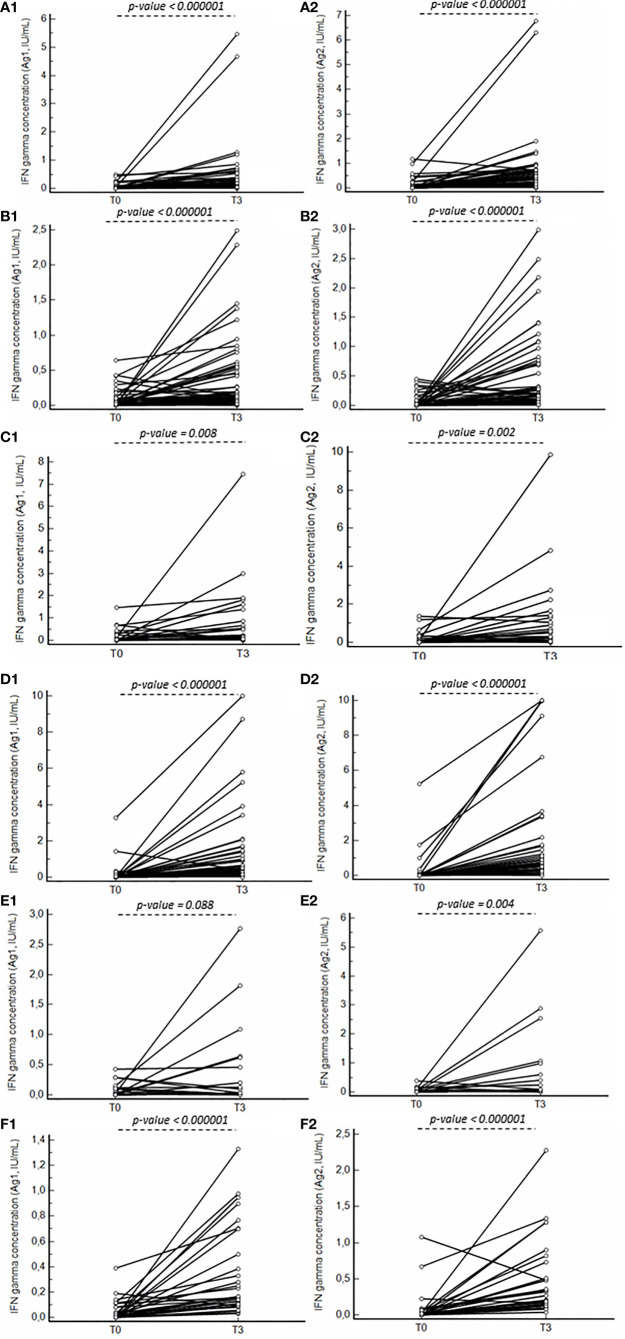
Line graphs presenting the difference in IFN gamma concentration for Ag1 (1) and Ag2 (2) between T0 and T3 for the healthy control group **(A)**, primary immunodeficiency patients **(B)**, PLWH **(C)**, neurology **(D)**, rheumatology **(E)** and CKD **(F)** patient groups. P-values indicating significant differences between the median concentrations are reported.

**Figure 3:**
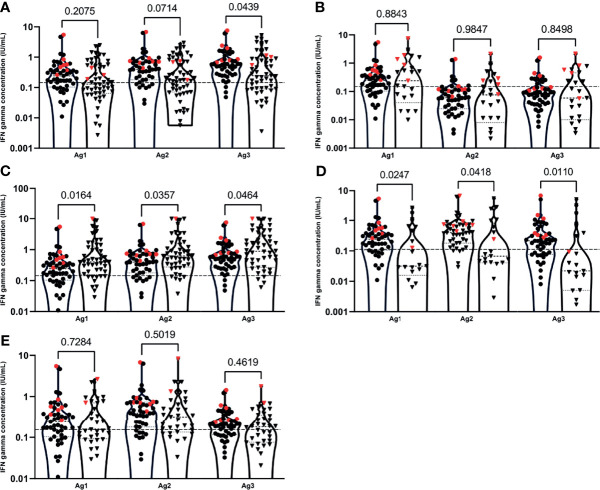
IFN gamma concentrations after stimulation of T-cells with the different antigen pools (Ag1, Ag2 and Ag3) between the healthy control group (●) and patient groups (▼): primary immune deficiency patient group **(A)**, PLWH **(B)**, neurology **(C)** rheumatology **(D)** and CKD **(E)** patient groups at T3. Patients with a documented Sars-CoV-2 infection are indicated with red symbols. Cellular responses were assessed by measuring interferon (IFN) gamma (IU/mL) by the QuantiFERON SARS-CoV-2 test on the Liaison XL analyser. Plots represent the median, 25th and 75th percentiles. Outliers were determined by 1.5 time IQR. Statistical significance was calculated by Mann-Whitney U test. Significance was defined as a p-value < 0.05.

At a cut-off positivity of 0.15 IU/mL, not all patients in the healthy control group exhibited IFN gamma positivity (**Table 3**) at T3. In all patient groups, a significant increase in IFN gamma concentration was observed between T0 and T3 for Ag1 and Ag2, except for the rheumatologic patient group for Ag1 (**Figure 2**). No significant differences in IFN gamma concentrations were observed at T0 between the healthy control and different patient groups for Ag1 and Ag2. Within each patient group, no significantly higher IFN gamma concentrations were observed after stimulation with the different antigens at T3, except between Ag1 and Ag 3 for the healthy control, neurologic and CKD patient groups, and between Ag1 and Ag2 for the healthy control group (**Figure 3**).

**Table 3:** Cellular immune response against each antigen (cut-off: 0.15 IU/mL) for each patient group at T3. Results are presented as number of positive patients per total number of patients at T3.

Patient group	Ag1		Ag2		Ag3		Number of patients without response to any antigen
	Positivity	Median (IU/mL, min-max)	Positivity	Median (IU/mL, min-max)	Positivity	Median (IU/mL, min-max)	
**Healthy controls**	35/51 (68.6%)	0.24 (0.00 – 5.47)	39/51 (76.5%)	0.40 (0.00 – 6.77)	45/51 (88.2%)	0.56 (0.00 – 7.57)	6/51 (11.8%)
**PID**	26/55 (47.3%)	0.13 (0.00 – 2.49)	33/55 (60.0%)	0.17 (0.01 – 2.99)	35/55 (63.6%)	0.24 (0.00 – 5.29)	18/55 (32.7%)
**PLWH**	14/25 (56.0%)	0.24 (0.00 – 7.44)	16/25 (64.0%)	0.49 (0.00 – 9.85)	17/25 (68.0%)	0.39 (0.00 – 9.97)	8/25 (32.0%)
**Neurology **	38/50 (76.0%)	0.40 (0.00 – 9.99)	41/50 (82.0%)	0.63 (0.00 – 9.99)	44/50 (88.0%)	0.89 (0.00 – 9.99)	5/50 (10.0%)
**Rheumatology **	7/21 (33.3%)	0.03 (0.00 – 2.76)	8/21 (38.1%)	0.07 (0.00 – 5.57)	9/21 (42.9%)	0.10 (0.00 – 6.11)	12/21 (57.1%)
**CKD**	19/36 (52.8%)	0.16 (0.00 – 2.65)	26/36 (72.2%)	0.32 (0.00 – 8.34)	27/36 (75.0%)	0.18 (0.00 – 9.98)	5/39 (12.8%)

PID, primary immunodeficiency; PLWH, patients living with HIV; CKD, Chronic kidney disease.

Median IFN gamma concentrations after T-cell stimulation with Ag1, Ag2 and Ag3 were significantly lower in the PID (except for Ag1) and rheumatologic patients at T3 as compared to the healthy controls (**Figure 3** and **Table 3**). In PLWH and CKD patients, no significant lower median differences compared to the healthy controls were observed. Remarkably, significantly higher median IFN gamma concentrations were observed in the neurologic patient group when compared to the healthy control patient group (**Figure 3** and **Table 3**).

Within the rheumatologic patient group, IFN gamma concentrations were lower than the cut-off in more than half of the patients at T3 (12/21; 57.1%) (**Table 3**). No statistical differences in cellular immune responses were observed between the group of patients that received BCDT as compared to the group that did not receive anti-CD20 for all 3 antigens in this patient group.

Within the PID and PLWH patient groups, no significant correlation between the lymphocyte CD4-count and IFN gamma concentration for all three tested pools of antigens was observed.

### Correlation Humoral and Cellular Immune Response

**Figure 4:**
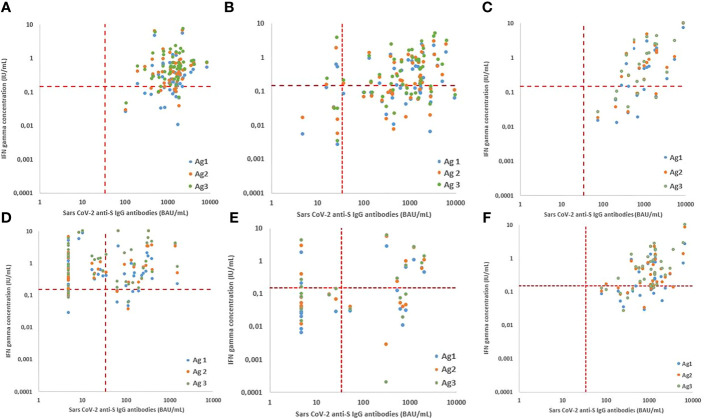
Relationship between cellular and humoral immune response after vaccination with Pfizer BNY162b2 for the healthy control **(A)** and different patient groups: primary immune deficiency patient group **(B)** PLWH **(C)**, neurology **(D)** rheumatology **(E)** and CKD **(F)** patient groups at T3. The relationship is presented for antigen 1 (blue), antigen 2 (orange) and antigen 3 (green). Cellular responses were assessed by measuring interferon (IFN) gamma (IU/mL) by the QuantiFERON SARS-CoV-2 test on the Liaison XL analyser and Humoral responses assessed by the Liaison^®^ SARS-CoV-2 TrimericS IgG chemiluminescent immunoassay on the Liaison XL. Correlation was calculated by using Spearman’s correlation coefficients.

SARS-CoV-2 IgG anti-S antibodies and QuantiFERON levels positively correlate significantly in the PID, PLWH and CKD patient groups for all 3 antigens (**Table 4** and **Figure 4**). In the healthy control group, a significant correlation was observed only for antigen 3 and in the rheumatologic patient group, a significant correlation was observed only for antigen 1 (**Table 4**). No significant correlation between humoral and cellular immune responses were observed in the neurologic patient group (**Table 4** and **Figure 4**).

**Table 4:** Spearman Rank correlation coefficients and significance levels between humoral and cellular immune response for the different tested antigens at T3 within each patient group. Significant correlations (p-value > 0.05) are indicated in bold.

Patient group	Ag1	Ag2	Ag3
**Healthy controls**	0.265	0.224	**0.301**
	(*p = 0.065*)	(*p = 0.126*)	**(*p = 0.036*)**
**PID**	**0.300**	**0.379**	**0.335**
	**(*p = 0.028*)**	**(*p = 0.004*)**	**(*p = 0.014*)**
**PLWH**	**0.585**	**0.532**	**0.592**
	**(*p = 0.004*)**	**(*p = 0.013*)**	**(*p = 0.004*)**
**Neurology **	0.121	0.215	0.112
	(*p = 0.413*)	(*p = 0.146*)	(*p = 0.450*)
**Rheumatology **	**0.480**	0.326	0.315
	**(*p = 0.028*)**	(*p = 0.161*)	(*p = 0.176*)
**CKD**	**0.426**	**0.440**	**0.566**
	**(*p = 0.012*)**	**(*p = 0.009*)**	**(*p = 0.0004*)**

PID, primary immunodeficiency; PLWH, patients living with HIV; CKD, Chronic kidney disease.

## Discussion

This evaluation of humoral and cellular immune response in a large cohort of patients diagnosed with immune deficiency disorders reveals, depending on the patient group, incomplete and/or delayed humoral and cellular immune responses up to 3 months after administration of the second dose of the BNT162b2 vaccine.

Humoral immune responses to SARS-CoV-2 are mediated by antibodies that are directed to viral surface glycoproteins, mainly the spike (S) glycoprotein and the nucleocapsid (N) protein. While the diagnostic performance of assays using RBD as an antigen have shown better results compared to spike and nucleoprotein antigens, the trimeric form was shown to be associated with greater sensitivity for the detection of SARS-CoV-2 IgG antibodies in human samples (26–29). In our study, almost 8% of the patients had positive IgG anti-S antibodies at T0. As can be deduced from **Figure 1**, these patients had higher antibody concentrations at the different study visits, confirming previous reports that prior SARS-CoV-2 infection results in improved humoral and cellular responses after first vaccine dose (30). Overall, the humoral immune response in the PLWH and CKD patient groups was acceptable and is comparable to responses that has been reported in health care workers (31). In contrast, responses were impaired in primary (PID) and secondary immunocompromised patients with underlying neurological and rheumatologic conditions. Also, the decay in antibody response between T2 and T3 in the PID and rheumatologic patient groups is larger compared to the other groups, which justifies additional booster vaccinations for this specific patients groups.

Conventional T-cells have an important role in long lasting protection conferred by immune memory: CD8+ cytotoxic T lymphocytes blunt virus replication by amongst others, secretion of immunostimulating cytokines including IFN gamma; CD4+ T helper on the other hand elicit a multiplicity of functions key to coordinating and regulating antiviral immunity (32). In the present study the QuantiFERON Sars-CoV-2 interferon gamma releasing assay (IGRA) was used for evaluation of cellular immunity. In contrast to other techniques including intracellular-cytokine staining or activation induced makers by flow cytometry and variation of the ELISPOT-assay which measures mainly IFN gamma concentrations, the tedious process of lymphocyte isolation, washing and counting is not required. In addition, the volume required for IGRA assays are lower and results can be interpreted objectively based on a predefined cut-off. As this assay can be applied to a large number of samples producing results within 24 h, this IGRA tests are particularly suitable for application in clinical laboratories. On contrary, stimulation by specifically chosen peptide pools is not possible using these T-SPOT tests. Whether this assay is equally sensitive for evaluation of cellular immune response after SARS-CoV-2 vaccination in different patient populations needs to be further determined (33).

Independent of the patient group, the Quantiferon SARS-CoV-2 assay detected an augmented cellular response in most of the Ag2 tubes compared to the Ag1 tube, indicating that both CD4+ and CD8+ T-cells contribute to the cellular response detected. Similarly, it has been reported that the BNT162b2 vaccine elicits strong CD4+ and CD8+ responses (34, 35). Our results extend the results of previous studies in health care workers that the highest IFN gamma concentrations were observed after stimulation with Ag3. This may be due to the selection of peptides included in the Ag3 tubes, as all subjects received a spike-based vaccine (34, 35).

These differences were only significant between Ag1 and Ag3 in the healthy control, neurologic and CKD patient groups. More than half of the patients in the rheumatologic patient group had negative IFN gamma concentrations after stimulation with any of the antigens. These observations may be explained by chronic T-cell suppression therapy in this patient group as well as other concomitant therapies. Remarkably, in the healthy control group, IFN gamma concentrations after stimulation with Ag3 were undetectable in almost 12% of the patients. As IFN gamma immune responses following vaccination are associated with certain class I and class II human leucocyte antigen (HLA) alleles, these results need to be interpreted with caution (32, 36). Future studies are needed to better understand the complex interplay between cytokines and the role of HLA gene polymorphisms in the outcome of SARS-CoV-2 immunity among populations with various HLA allelic distributions.

Significant positive correlations between humoral and cellular immune responses were obtained in the healthy control, PLWH and CKD patient groups. These results are of value as cellular immunity is not routinely determined in the clinical lab. In secondary immunocompromised patients with rheumatological and neurological conditions on BCDT whether or not in combination with chronic T-cell suppression therapy, correlation between humoral and cellular immunity was poor and not significant.

Studies that describe humoral and cellular immune responses to SARS-CoV-2 vaccination in immunocompromised populations, especially in patients diagnosed with PID, are scarce (22, 23, 37). In our study, we included 57 patients diagnosed with a variety of PID disorders. The majority of these patients were able to respond to the vaccine with antibody positivity above the threshold (94.7%) after 2 vaccine doses. Further, a significant but weak correlation between humoral and cellular response in PID patients was observed (**Figure 4** and **Table 4**), which may be attributed to the patients with combined immunodeficiency. Besides IRT, also lymphopenia as well as BCDT (rituximab) were associated with deficient antibody responses in the whole group.

Our results in the PLWH confirm previous reports showing that BNT162b2 mRNA vaccine induces good antibody responses and that the level of binding antibodies is not significantly different from that observed in healthy volunteers (14, 15, 38). We extend the results of previous studies by demonstrating that also the cellular immune response in PLWH is not statistically different compared to healthy donors up to 3 months after administration of the second vaccine dose. These results are in accordance with the results of Woldemeskel *et al*, who demonstrated a robust humoral and cellular immune responses 7 to 17 days after administration of the second vaccine dose in PLWH (16). In addition to these findings, we demonstrated that PLWH with CD4 T-cell counts lower than 350/µL at inclusion have the same robust humoral and cellular immune responses.

It has been described that humoral immune response in patients treated with BCDT (rituximab, ocrelizumab) therapy is poor (39, 40). These results are in accordance with the humoral response observed in the rheumatologic and neurologic patient groups in our patient cohort. Despite poor cellular responses in the rheumatologic patient group, almost all of the neurologic patients generated robust CD4 and CD8 cellular responses to SARS-CoV-2 mRNA vaccination suggesting that vaccinating subjects on BCDT is likely to provide some level of cellular immunity to SARS-CoV-2 in absence of humoral immune response. In contrast to the rheumatologic patient group, significantly higher IFN gamma concentrations were observed in the neurologic patient group after stimulation of CD4/CD8+ T-lymphocytes with all three antigens compared to the healthy control group. These findings could be explained by the concomitant administration of chronic T-cell suppressive therapy to the rheumatologic patients included in our study (41). Also, the difference in age between both patient groups as well as the underlying immune disorder of the patients included in the rheumatologic patient group may explain the poor cellular response in this specific patient group. The neurologic group forms a more homogeneous group, less confounded by concomitant immune therapies, with the majority being MS patients all treated only with BCDT. The increased cellular response also could be compensatory for the impaired humoral immune response in the neurologic patient group. One limitation of our study was the relative low number of patients included in the rheumatologic patient group. Therefore, it was difficult to draw hard conclusions on cellular immunity in patients treated with T-cell inhibition or suppression therapies.

Most of the patients included in the CKD patient group were diagnosed with CKD stage G3b and G4 (35/39, 89.7%). Overall, 94.9% and 100% of the patients showed a humoral immune response 21 to 28 days after the first dose and 10 to 14 days after the second dose, respectively. Compared to the healthy control group, the serologic response at T1 is decreased. On the contrary, peak antibody levels in our patient group occurred 4 to 5 weeks after the first vaccine dose, thereby reaching the same antibody concentrations as in the healthy control group. These findings contrast with the serologic responses in haemodialysis patients, which are blunted and delayed (11, 25, 42–45). The median IFN gamma concentration as well as the proportion of patients exceeding the threshold for positivity was not significantly different compared to the healthy controls. These results show that in contrast to the results of Van Praet *et al*, who demonstrated a significant difference in spike-specific CD4 and CD8 T-cell responses compared to healthy volunteers, pre-dialysis patients are less immunocompromised. As the same assay was used to assess cellular immune response, these differences probably can be attributed to the CKD-stage and difference in median age of the haemodialysis patients included in the study. Despite reduced immunity and mild to moderate lymphopenia of our CKD patient group, an adequate humoral and cellular immune response was observed. These results are in agreement with other immunization studies in CKD patients in which it has been reported that higher GFR levels are more likely to respond to hepatitis B vaccination programs (46).

Currently, effective as well as cost efficient vaccination policy is being discussed in the scientific healthcare community worldwide. Our study shows that there are significant differences in the humoral and/or cellular response after vaccination with two doses of the BNT162b2 vaccine in different clinical groups of severely immunocompromised patients. These findings imply that more tailored risk stratification of necessity of additional vaccine doses and follow-up strategies is feasible depending on the presence as well as evolution of immune responses in the specific groups of patients. Laboratory measurements of post-vaccination immunity in certain groups of patients can serve as a practical tool for the guidance of further protective measures to be taken

In conclusion, immune response after SARS-CoV-2 immunization is impaired in some patients with acquired and inherited immune deficiency disorders. Whether humoral, cellular or both immunogenicity is delayed depends on the patient group, therapy and individual risk factors. In contrary, immune response in PLWH and CKD patients included in our were comparable with the results obtained in the healthy control group. Future studies could address the longevity of the humoral and cellular immune responses 6 to 12 months after primary vaccination. These studies will allow to determine the longevity and effect of additional booster vaccination in patients with primary and secondary immunocompromised patients. The obtained results on humoral and cellular immune responses in PID, neurology and rheumatology patients may be used to adapt current guidelines on the relationship between COVID-19 vaccination and certain therapeutics in specific clinical groups as well as well as the utility of measurements of humoral and cellular immune responses for the guidance of individual immunocompromised patients.

## Data Availability Statement

The original contributions presented in the study are included in the article/**Supplementary Material**. Further inquiries can be directed to the corresponding author.

## Ethics Statement

The studies involving human participants were reviewed and approved by Ethical committee of the Ghent University Hospital (Ref. B6702021000426). Written informed consent to participate in this study was provided by the participants’ legal guardian/next of kin.

## Author Contributions

MO, M-AS, GL, FH, LH, TK, SL, PJ and EP designed the study. EP gained grants for this evaluation. SH, LN, LH and KC collected the clinical data and coordinated the study visits. MO and EP analysed the data and wrote the article. M-AS, SH, GL, SA, MC, FH, LN, LH, KC, TK, SL, WB, PJ, BV and EP read the article carefully and made corrections were appropriate. All authors contributed to the article and approved the submitted version.

## Funding

This project has received sponsoring from UZ Gent COVID-19 Foundation.

## Conflict of Interest

The authors declare that the research was conducted in the absence of any commercial or financial relationships that could be construed as a potential conflict of interest.

## Publisher’s Note

All claims expressed in this article are solely those of the authors and do not necessarily represent those of their affiliated organizations, or those of the publisher, the editors and the reviewers. Any product that may be evaluated in this article, or claim that may be made by its manufacturer, is not guaranteed or endorsed by the publisher.

## Supplementary Material

Supplementary Figure 1 Box-and-Whisker plots of anti-SARS-CoV-2 IgG antibodies at T0, T1, T2 and T3 time points between patients receiving intravenous immunoglobulin substitution therapy (IVIG) (▼) and patient not receiving IVIG (●) within the primary immunodeficiency patient group **(A)** and between patients receiving rituximab (▼) an not receiving rituximab (●) within the rheumatology patient group **(B)**. Boxes represent the median, 25th and 75th percentiles, whiskers show the largest and smallest non-outlier values. The patients indicated in red are outliers. Outliers were determined by 1.5 time IQR. Statistical significance was calculated by Mann-Whitney U test. Significance was defined as a p-value < 0.05. *IRT, Immunoglobulin replacement therapy*.

## Abbreviations

Ag, antigen; SARS-CoV-2, severe acute respiratory syndrome coronavirus 2; PID, primary immune deficiency; PLWH, people living with HIV; HIV, human immunodeficiency virus; CKD, Chronic Kidney Disease; GFR, glomerular Filtration Rate; PCR, polymerase chain reaction; CLIA, chemiluminescent immunoassay; BAU, binding activity units; RBD, receptor binding domain; NTD, N-terminal domain; N, nucleocapsid; S, Spike; CMIA, chemiluminescent micro-particle immunoassay; IFN, interferon; KDIGO, Kidney Disease Improving Global Outcomes; BCDT, B-cell depletion therapy; NMO, neuromyelitis optica; MS, multiple sclerosis; IRT, Immunoglobulin Replacement Therapy; NA, not applicable; GPA, granulomatosis with polyangiitis; RITUX, Rituximab; DMARD, Disease modifying anti-rheumatic Drug; CORTIC, Corticosteroid; METHO, Methotrexate; OCRE, ocrelizumab; Y, Years; ID, immune deficiency, AB, antibody; AKI, acute kidney disease; CAKUT, Congenital Abnormalities of the kidney and urinary tract; IUIS, International Union of Immunological Societies; IS, immune suppression; SD, standard deviation; IGRA, interferon gamma releasing assay.
