# Supplementary figures and images for: Evaluation of Humoral and Cellular Responses in SARS-CoV-2 mRNA Vaccinated Immunocompromised Patients
Source: Front Immunol. 2022 Mar 22;13:858399. doi: 10.3389/fimmu.2022.858399 (PMC8988283; doi:10.3389/fimmu.2022.858399)

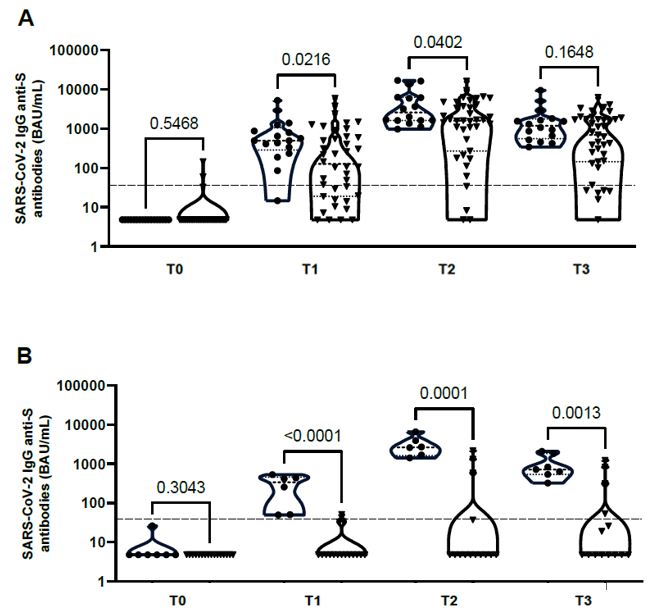

Supplement: Supplementary Figure 1 — Box-and-Whisker plots of anti-SARS-CoV-2 IgG antibodies at T0, T1, T2 and T3 time points between patients receiving intravenous immunoglobulin substitution therapy (IVIG) (▼) and patient not receiving IVIG (●) within the primary immunodeficiency patient group (A) and between patients receiving rituximab (▼) an not receiving rituximab (●) within the rheumatology patient group (B). Boxes represent the median, 25th and 75th percentiles, whiskers show the largest and smallest non-outlier values. The patients indicated in red are outliers. Outliers were determined by 1.5 time IQR. Statistical significance was calculated by Mann-Whitney U test. Significance was defined as a p-value < 0.05. IRT, Immunoglobulin replacement therapy. [file Image_1.tif]
